# Scalable genotyping of microbial colonies

**DOI:** 10.1099/mgen.0.001378

**Published:** 2025-03-19

**Authors:** Arnold Chen, Nkazi Nchinda, Nate J. Cira

**Affiliations:** 1 Meinig School of Biomedical Engineering, Cornell University, Ithaca, 14853, NY, USA; 2 Harvard Medical School, Harvard University, Boston, 02115, MA, USA

**Keywords:** colony PCR, genotyping, microbes, primer multiplexing

## Abstract

The sequence of the 16S region is taxonomically informative and widely used for genotyping microbes. While it is easy and inexpensive to genotype several isolates by Sanger sequencing the 16S region, this method becomes quite costly if scaled to many isolates. High-throughput sequencing provides one potential avenue for obtaining 16S sequences at scale but presents additional challenges. First, DNA purification workflows for high-throughput sample preparation are labour-intensive and expensive. Second, cost-effective multiplexing and library preparation schemes are difficult to implement for many libraries on a single sequencing run. Therefore, we implemented a scalable protocol for isolate genotyping involving colony polymerase chain reaction (PCR) with simple cell lysis as well as a four-barcode indexing scheme that enables scalable multiplexing and streamlined library preparation by amplifying with four primers simultaneously in a single reaction. We tested this protocol on 93 colonies cultured from environmental samples, and we were able to ascertain the identity of ~90% of microbial isolates.

**Impact Statement**

Genotyping by sequencing parts of the genome is a powerful way to characterize microbes. However useful, this information can be impractical to obtain for large collections of isolates, since library preparation is a major bottleneck. Here, we present a method for 16S sequencing that involves creating amplicons directly from colonies using four indexed primers simultaneously in a single reaction. The end result is a streamlined workflow that enables scalable genotyping, resolving the challenge of library preparation throughput and allowing researchers to better capitalize on the capacity of current sequencers.

## Data Summary

Illumina sequencing data are available in the SRA repository with the accession number PRJNA1052305 at the link https://www.ncbi.nlm.nih.gov/sra/PRJNA1052305. Other sequencing data such as amplicon sequence variants and Sanger sequences are available in Tables S1, S2 and S3, available in the online Supplementary Material.

## Introduction

Growing microbial isolates allows them to be characterized and utilized. Libraries of microbial isolates are collected for many purposes, including the production of natural product metabolites for drug discovery [1], recapitulating representative microbial communities such as gut microbiomes [2], investigating organism ecology and evolution [3], cataloguing clinical isolates [4] and the growth and discovery of new microbes [5]. Frequently, isolates from such libraries need to be genotyped to, for example, identify organisms of interest [6], dereplicate clones [1], identify which clones have a gene of interest [7] and determine organism relatedness [3].

Genotyping a single gene for a single isolate is straightforward and is commonly accomplished with Sanger sequencing. Starting with a grown isolate, this process often involves microbial lysis, DNA purification, DNA amplification and product clean-up before Sanger sequencing and analysis. This can be accomplished at a reasonable cost for a few isolates but quickly becomes time-consuming and costly when scaling to large collections of isolates.

High-throughput sequencing generates far more sequenced bases per sequencing run than Sanger sequencing and is widely used to analyse many organisms at once in the study of microbial communities [8,10]. However, each sample must be prepared and made into a library independently, which is typically even more burdensome than preparing samples for Sanger sequencing. For a large collection of isolates, library production becomes a significant barrier.

Prior work has employed various strategies when designing libraries for microbial samples. These include incorporating spacer, linker and pad regions into primers [11,15], introducing clamping for selective amplification [12,16] and implementing multiplexing approaches such as primer tagging [17,18] and dual barcoding [19] to efficiently index samples.

Here, we developed a workflow for the streamlined production of high-throughput amplicon sequencing libraries. We show how this workflow enables scalable genotyping by sequencing the 16S V4 region of a library of microbial isolates.

## Methods

### Overview

**Fig. 1.**
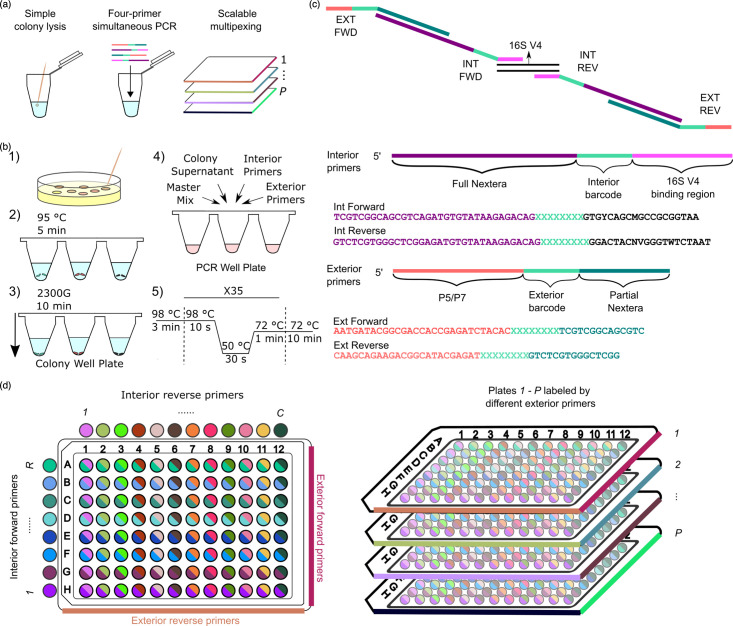
A schematic depicting the scalable genotyping workflow. (**a**) An overview of the features of the scalable genotyping workflow. (**b**) Each colony was picked from agar plates, placed in water, heated and then centrifuged. The supernatant was mixed into the PCR reaction mix containing master mix and both interior and exterior primers. The reaction was thermal-cycled. (**c**) Interior primers anneal to the 16S rRNA V4 region, and exterior primers bind to the Illumina flow cell. Each amplicon is accompanied by four primers: exterior forward (EXT FWD), interior forward (INT FWD), exterior reverse (EXT REV), and interior reverse (INT REV). Each primer contains an index, and the final amplicon contains four different indices. (**d**) The library indexing scheme encodes rows with interior forward primers and columns with interior reverse primers. One set of exterior primers was used throughout the whole well plate (left). The scheme is easily scalable to multiple well plates (right) by using exterior indices to label each plate.

We devised a scalable genotyping protocol (Fig. 1a) which features (1) colony PCR with simple lysis, (2) indexing conducive to scalable multiplexing across multiple well plates and (3) four-barcode indexing performed in one reaction, excluding the need for intermediate clean-ups and additional reactions. In this section, we describe our protocol in detail. In addition, we discuss the rationale behind our specific approach and the trade-offs compared to alternative methods.

### Sample collection, colony picking and colony freezer stocks

Ten environmental samples were collected from various locations near Cornell University, Ithaca, NY. Each sample was prepared with different dilution treatments, including undiluted, $10^{-1}$, $10^{-2}$ and $10^{-3}$ dilutions, and then plated on tryptic soy agar (TSA), Luria agar and 5% sheep blood in tryptic soy agar plates. Plates were incubated at room temperature for 3 days.

A total of 93 colonies were selected, with 31 colonies from each type of agar plate, being careful to exclude colonies that were in contact with another colony. In addition, we made freezer stocks of isolates to catalog them for future use.

### Colony lysis

The first step of most genotyping workflows is to prepare purified DNA from the sample, often using commercial kits or protocols that become costly and onerous to perform at a large scale. A more scalable approach is colony PCR [20], which involves processing microbial colonies without rigorous purification, instead using only simple procedures such as heating and centrifuging before downstream reactions.

Here, we adapted a colony PCR protocol [21]. The colony material was picked with pipette tips into wells of a 96-microwell plate with 80 µl of PCR-grade water (Invitrogen) (Fig. 1b). The microwell plate was sealed with PCR plate foil, heated at 95 °C for 5 min to crudely lyse the colonies and centrifuged at 2300 ***g*** for 10 min to pellet the cellular debris. The supernatant of each colony was used as a DNA template for the PCR. This approach, while unlikely to provide a high concentration of purified template DNA, avoids the cost and time associated with more involved sample preparation methods.

### Primer design

A single high-throughput sequencing run generates ample sequencing reads, allowing for cost-effective sequencing of numerous pooled samples. When pooling samples, a multiplexing scheme must be used to assign which sample each read originates from. Multiplexing is accomplished by introducing different indices into each sequencing library to encode the sample of origin. A simple scheme is to incorporate a different index for each sample. For a number of samples, *N*, the number of primers required is also *N.* In cases with many samples, buying a custom primer for each sample results in high costs.

Alternatively, combinatorial dual indexing can be used, where indices are repeated within the same row and column in a well plate format, and each sample is encoded by a unique row/column combination. This reduces the required number of primers to ~2$\sqrt{N}$, where *N* is the number of samples. For Illumina sequencing, this indexing scheme is most commonly implemented through i5/i7 indices but is susceptible to an undesirable phenomenon called index hopping [22], where indices are misassigned on Illumina sequencers. Index hopping is thought to arise when free adaptors containing i5 and i7 sequences are incorporated into the template by steps in the Illumina sequencing protocol [23]. Since libraries in the same row or column differ by only the forward or reverse index, misassigning either index will lead to the assignment of the read to the wrong sample.

**Fig. 2.**
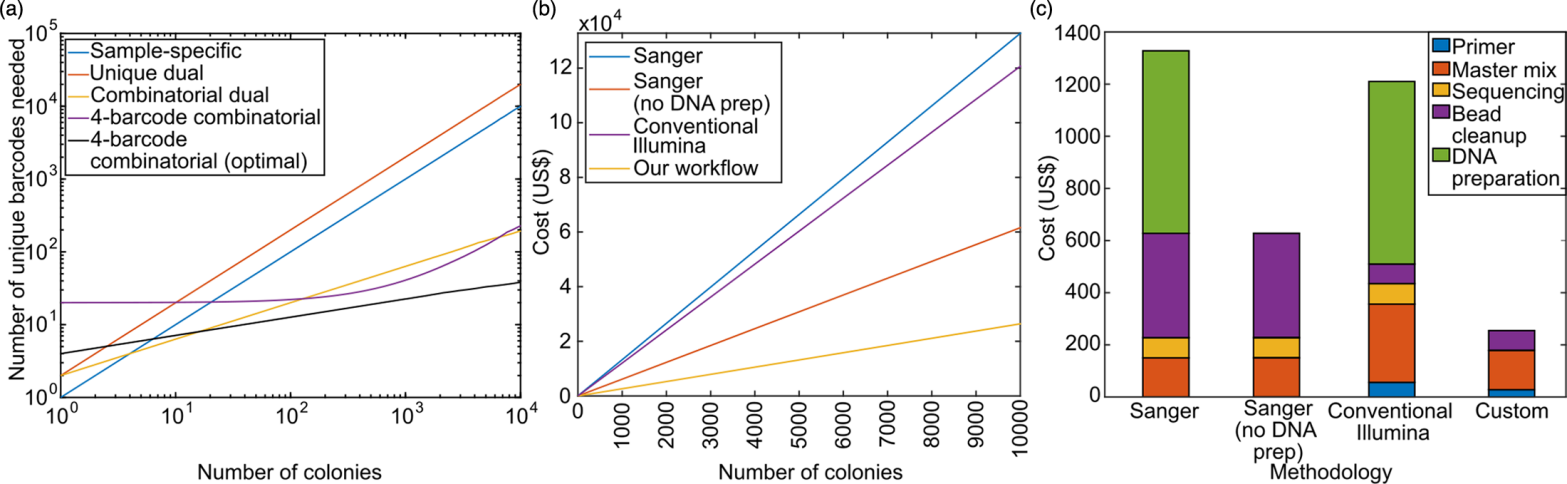
Comparison of sequencing and indexing workflows in terms of barcodes needed and overall cost. (**a**) Number of unique primer barcodes needed for a given number of colonies using 96-well plate formats. The sample-specific and unique dual indexing schemes scale linearly with colonies. The four-barcode combinatorial indexing scheme using the exterior barcodes as dual unique plate labels scales linearly with colonies for larger numbers of colonies but requires fewer barcodes than the sample-specific and unique dual index schemes. It is comparable to the combinatorial dual scheme in efficiency but does not suffer from index hopping (Figure S1). The optimal four-barcode combinatorial indexing scheme scales very efficiently, with the fourth root of the number of colonies. The difference between four-barcode combinatorial and four combinatorial (optimal) is in the way the plate is indexed. The four-barcode combinatorial assumes wells in the same row or column use the same barcode. The four-barcode combinatorial (optimal) is based on the $4\sqrt[{4}]{N}$ calculation, which reduces the total number of primers needed most efficiently, but wells in the same row or column may not share the same index, so it may be more tedious to keep track experimentally. (b) The cost of different microbial genotyping workflows comparing Sanger, Illumina and our custom workflow. (**c**) The estimated cost breakdown of different genotyping workflows per 100 isolates. See Table S8 for a detailed table of cost and explanations.

To address index hopping, a unique dual indexing scheme can be used, where each library uses two unique indices, requiring 2*N* total primers for *N* samples. If either the forward or reverse index is misassigned, this error can be inferred from the other index. However, the number of unique primers in this scheme becomes prohibitively expensive for large *N* (Fig. 2a).

To enable scalable multiplexing, yet avoid index hopping and avoid high costs associated with unique dual indexing, we opted for a four-barcode multiplexing scheme using four primers for each amplicon. The scheme includes exterior forward, exterior reverse, interior forward and interior reverse primers (Fig. 1c). The primers are referred to as ‘exterior’ and ‘interior’ because in the final amplicon, the interior primer sequence is situated in between the target DNA of interest and exterior primer sequence. Interior primers anneal to the target DNA of interest, while exterior primers contain the Illumina P5/P7 regions that allow binding to the flow cell. The exterior primers overlap with the Nextera region of the product created by the interior primers, resulting in a longer amplicon with all the necessary sequence regions for indexing and sequencing. To allow for multiplexing, we incorporated 8 bp barcode sequences to all four primers.

We used four indices for each amplicon using interior indices to label the well location and exterior indices to label the plate. We barcoded a 96-microwell plate with unique pairwise combinations using 8 different interior forward indices along the rows and 12 different interior reverse indices along the columns (Fig. 1d) (Table S4). One exterior forward and one exterior reverse index were used to label the plate. This version of a four-barcode scheme requires ~(2P+R+C) unique primers, where *P* is the number of plates and *R* and *C* are the number of rows and columns in each plate. Across multiple 96-well plates, this requires $\sim (\frac{2N}{96}+20)$ total primers, which scales better than the sample-specific and unique dual indexing schemes. Notably, this scheme avoids index hopping because the exterior primers, those containing the i5 and i7 indices, are dual unique. Theoretically, this scheme can be very primer-efficient, requiring only $4\sqrt[{4}]{N}$ primers for *N* samples, though this would often require inconvenient index layouts with respect to processing in well plates. The term $4\sqrt[{4}]{N}$ is calculated from the fact that each well uses four kinds of primers: interior forward, interior reverse, exterior forward and exterior reverse primers. The rule of product states that the number of ways to index *N* wells using these four primers is the product of the number of options for each primer. This product is maximized when the number of options for each primer is the same, giving *N* possible wells to index for $4\sqrt[{4}]{N}$ total primers or $\sqrt[{4}]{N}$ primers for each of the four primer types.

For genotyping, we selected the ~254 bp 16S V4 region. The 16S region and its subregions are commonly used to infer phylogeny. The throughput and amplicon length of the Illumina sequencing technology make it suitable for sequencing subregions with such lengths. Theoretically, the maximum length that a region can have that is still suitable for our workflow is constrained by Illumina’s 300×2 amplicon sequencing chemistry. However, while the theoretical maximum is 600 bp, there are several factors to consider. Sufficient overlap between the forward and reverse reads is desirable to allow proper merging of reads during sequence analysis. Our workflow uses interior primers, which means a portion of the read is allocated towards the interior primer barcode and the known sequence that binds to the region of interest. Thus, other 16S subregions with similar or shorter lengths as 16S V4 would likely be suitable choices for the choice of sequencing region. Longer regions could be possible if compromises are made on the above (e.g. less read overlap and shorter primer regions).

To target the 16S V4 region, we incorporated primer sequences 505 F [24] and 806 R [25] for the forward and reverse interior primers, respectively. The target overlapping regions on these primers are easily modifiable to target other variable regions (e.g. amplification of the V3–V4 and V7–V9, as demonstrated in Figure S2) (Table S5) while still maintaining the four-barcode multiplexing capabilities.

### Library preparation and amplicon sequencing

Early barcode schemes have demonstrated amplification using two primers, involving sample-specific single-barcode indexing [26,27]. This is later extended to two-barcode indexing, with one barcode on each of the two primers [19,28, 29], which enables combinatorial indexing.

Prior work has demonstrated these barcode schemes involving four primers. This includes a sample-specific single-barcode scheme through sequential amplification, typically requiring more than one round of thermal cycling with intermediate treatment or clean-up steps [30,32]. This sequential amplification can incorporate two-barcode indexing [33] as well. An advantage of using four primers becomes apparent when incorporating four-barcode indexing instead [14,34,36], which enhances the combinatorial capabilities of the scheme. Other works have shown simultaneous addition and amplification with four primers, but primarily with two-barcode indexing through dual unique barcodes [37] and dual identical barcodes [38]. In our approach, we implemented a four-barcode scheme where we simultaneously added all four primers, which contained all the necessary indices and adaptors, into the reaction mix under one thermal cycled reaction. Our approach omits the need for additional reactions, thus reducing costs and avoiding the parallel clean-up between different rounds of amplification. This allows us to reap the benefits of scaled multiplexing without the effort of additional reactions.

Each 25 µl reaction volume contains 2 µl of colony supernatant, 0.05 µM of each interior primer, 0.45 µM of each exterior primer and 12.5 µl of master mix (2X Phusion High-Fidelity PCR Master Mix, New England Biolabs). The high ratio of exterior to interior primers helps ensure that the dominant product is the desired full-length amplicon. The presence of some interior amplicon is not problematic, as it will not sequence without Illumina adaptors. The reaction was thermal-cycled with the following conditions: initial denaturation at 98 °C for 3 min, followed by 35 cycles of denaturation (98 °C for 10 s), annealing (50 °C for 30 s) and extension (72 °C for 60 s) and a final extension step at 72 °C for 10 min.

PCR products were pooled and then purified with AMPure XP beads (Beckman Coulter) at a 0.7× bead/sample ratio. Sequencing was performed with a portion of the Illumina MiSeq v3 kit to obtain ~800 000 paired-end reads, each 300 bp, at the Cornell Institute of Biotechnology (Ithaca, NY).

### Bioinformatic analysis

Raw reads were demultiplexed by the interior indices to assign the well location. DADA2 [39] was used to filter the reads using default parameters. Excluding the barcode and binding regions, forward reads were further truncated to 180 bp, while reverse reads were truncated to 120 bp, based on an initial assessment of per base sequence quality. Reads were merged to create amplicon sequence variants (ASVs) (Table S1), and abundances were tabulated based on the well location (Table S6). ASVs were deemed representative of a well if the ASV read abundance in the well passed a minimum read threshold of 15 reads and a minimum proportion of 0.1 of the total read abundance of that well. We determined the taxonomy associated with each ASV by aligning reads with the silva [40] SSU 138.1 database using VSEARCH [41]. A phylogenetic tree was created by first performing multiple sequence alignment through muscle [42] then using mega11 [43] to construct maximum likelihood trees with 100 bootstrap replicates.

### Sanger sequencing and analysis

To confirm the identity of organisms obtained through Illumina sequencing, wells with two or more representative ASVs and four additional wells were Sanger-sequenced. Freezer stocks corresponding to these wells were plated and grown on agar plates of the corresponding media type. The colonies were purified (Qiagen DNeasy Powersoil Pro kit). Amplification was conducted using interior primers (Table S4), and product clean-up was performed (AMPure XP beads). The products were submitted for Sanger sequencing at the Cornell Institute of Biotechnology. The Sanger chromatograms were examined, and sequences were aligned with ASVs to identify matches.

## Results and discussion

**Fig. 3.**
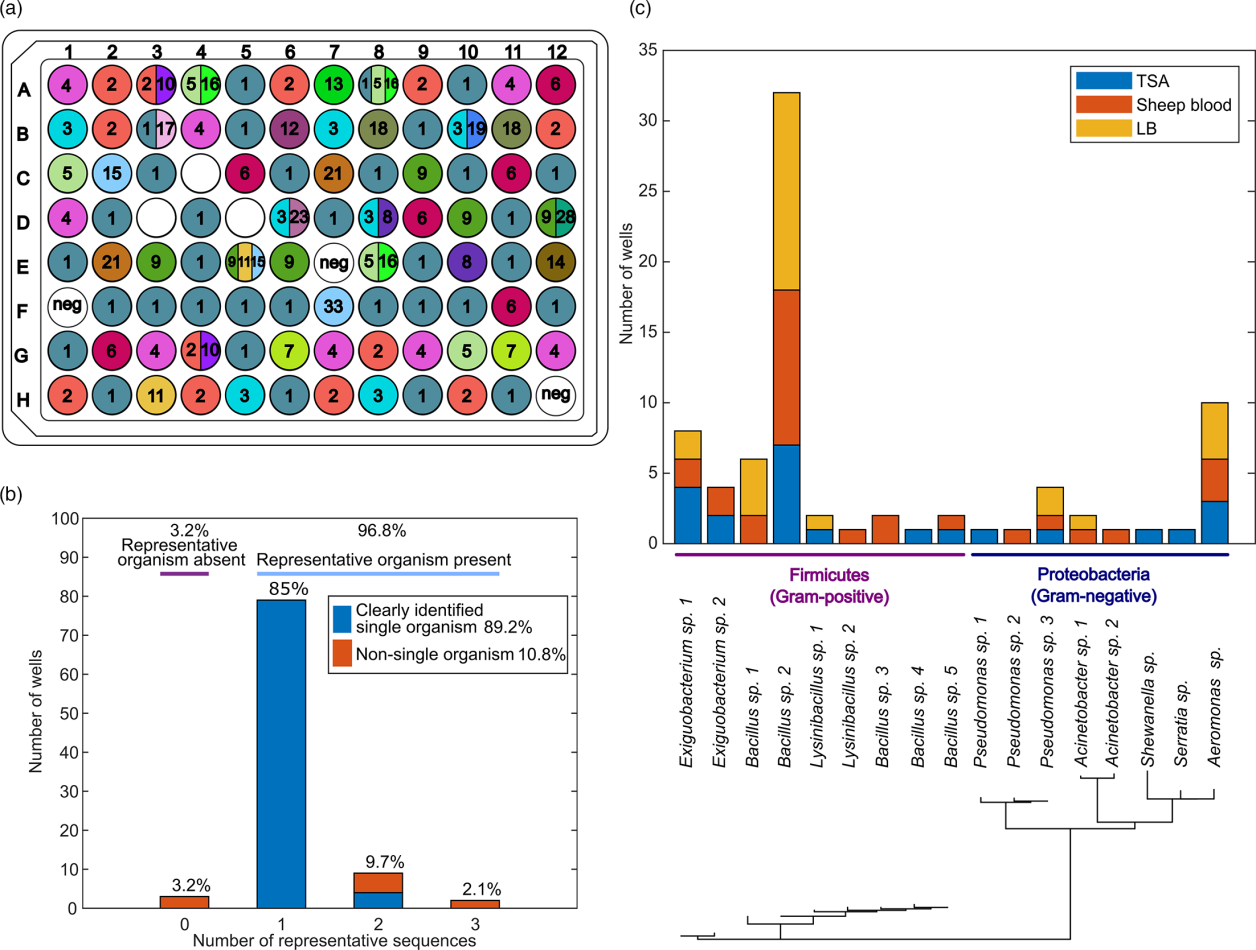
Representative ASVs and identified organisms through the implementation of the scalable workflow. (**a**) Representative ASVs in each well. Each number and colour indicates a unique ASV. White wells indicate no representative ASV was detected. (**b**) The proportion of wells that contained between 0 and 3 representative ASVs, coloured and labelled with the proportion of wells that contained representative organisms and the proportion of wells where we were able to clearly identify the organism’s taxonomy. (**c**) The assigned genera, media of isolation and phylogenetic tree of the representative ASVs.

To assess the performance of our workflow, we mapped the representative ASVs to the well positions (Fig. 3a). Overall, 96% of the wells amplified successfully and had at least one representative ASV (Fig. 3b). We taxonomically identified these organisms from environmental samples, finding organisms from two phyla: *Firmicutes* and *Proteobacteria*. There were eight unique genera, of which three were Gram-positive, *Exiguobacterium, Bacillus* and *Lysinibacillus* and five were Gram-negative, *Pseudomonas, Acinetobacter, Shewanella, Serratia* and *Aeromonas* (Fig. 3c).

Next, we categorized the wells based on the number of ASVs they mapped to and further investigated select wells through Sanger sequencing. Out of 96 wells, the three negative controls did not yield any representative ASVs, as expected. Of the 93 wells with isolates, 79 wells matched to only one representative ASV, providing clear taxonomic identification of those organisms. For confirmation, we replated and Sanger-sequenced freezer stocks from four wells (Table S2), which indeed matched with the original ASV sequences. Fourteen ASVs appeared in multiple wells, which is not unexpected; when sequencing multiple colonies per sample, it is likely that some colonies will have the same V4 sequence. In the remaining 14 wells, 11 wells had more than one representative ASV mapped to each (Fig. 3b). We investigated the identities of organisms in these 11 wells through regrowth of the freezer stock and Sanger sequencing. We obtained sequences from 8 out of the 11 wells (Table S2), but poor amplification and failed Sanger sequencing prevented us from obtaining sequences from the remaining three wells. Out of the eight sequences, all sequences matched one of the ASVs in the corresponding wells (Table S7), supporting the validity of the ASVs. In four out of these eight wells, the multiple ASVs within a well only differed by 1 bp, which was also observed in Sanger sequencing as a mixed signal at the same base pair discrepancy (wells A3, A4, B10 and E8) (Figure S3). This may be attributed to organisms possessing intragenomic variation, which is well documented for the 16S rRNA gene [44,45]. Three wells contained non-amplified organisms, potentially due to fungal colonies without a 16S region, PCR inhibitors or the colony’s susceptibility to lysis [46,47]. These results demonstrate the utility of this workflow to properly identify the taxonomy of a collection of microbial isolates. Overall, 90 out of 93 wells (96.8%) yielded representative sequences, and 83 out of 93 wells (89.2%) indicated a single clear organism.

In addition to the successful identification of isolates, cost is a key aspect to consider for a scalable workflow. We compared the cost of four workflows: conventional Sanger sequencing, Sanger sequencing with no DNA preparation, conventional Illumina sequencing with two-step PCR and our custom approach (Fig. 2b) (Figure S4). Note that we consider the costs on a per-colony basis. In certain cases, there may be an initial upfront cost involved with obtaining the required reagent and primer stock at a volume larger than required for the experiment. However, the excess reagent stock can often be used in future experiments, and average cost calculation becomes especially applicable in scaled-up experiments.

In all four workflows, the cost scales linearly with the number of colonies under the above assumptions. Conventional Sanger sequencing has the highest per-colony cost, followed closely by conventional Illumina sequencing. Sanger sequencing with no DNA preparation has roughly half the per-isolate cost compared with these two methods. Our custom workflow has less than half the per-isolate cost compared with the other methods.

The contribution to the total cost comes from various factors, including DNA preparation, reagents (primers and master mix), sequencing and purification of PCR products (Fig. 2c) (Table S8). One key factor that led to the reduced cost of our scheme is the omission of DNA preparation, which accounts for a major fraction of the cost in the other two schemes. Another benefit is that only one bead purification is required for our scheme. In the other three Sanger and Illumina workflows, bead purification is typically performed on each reaction in parallel after the initial amplification and thus bead purification costs scale linearly with the number of samples. While our custom workflow has a higher primer cost than both Sanger workflows, the large cost reduction in other factors produces a lower overall cost than the Sanger and conventional Illumina workflows. In addition to the reagent costs considered above, our custom workflow also reduces the time and labour required by omitting DNA preparation, parallel bead purification of separate products and secondary PCR reactions. The master mix remains a major contributor to our custom scheme’s cost, so miniaturization of reagent volumes is key to further reducing the cost of scaled microbial genotyping workflows. We demonstrate this miniaturization along with the scale-up to multiple well plates in a follow-up work [48].

Overall, this workflow provides easy genotyping of microbial isolates. However, there are still several limitations. First, while we have demonstrated the ability to largely bypass the DNA extraction step to save cost, this is performed on culturable microbes. Mixed communities from uncultured sources may have strong biases in lysis efficiency and are better suited to standard DNA extraction protocols. Second, here we sequence the 16S V4 region, which is ~254 bp. The method is not applicable to sequencing very long sequences such as whole genomes. It is possible to sequence longer amplicons on Illumina instruments, provided they are less than 600 bp, though with some compromises (see ‘Primer design’ in the ‘Materials and methods’ section), which would allow for sequencing with certain combinations of multiple variable regions. We have so far demonstrated amplification for the V4, V3–V4 and V7–V9 regions. Sequencing over 600 bp is possible through the use of other sequencing platforms (e.g. long-read sequencing), which can be adapted to our workflow by subsituting Illumina sequencing with other sequencing approaches. Third, our method is limited to amplifying the particular region(s) specified by the primers. Thus, if there are other organisms such as fungi in a collection of unknown microbes, then targeting the 16S region for amplification would miss those organisms since they will not be amplified.

In addition, we compare the success rate of our workflow with some in the literature. Note that several factors can cause different workflows to be compared on a biassed basis. More difficult sample types may result in a lower success rate for a workflow. Using more rigorous isolation or purification protocols will likely lead to a higher success rate; while it is advantageous in this regard, it will likely require higher costs. Nevertheless, they still offer some basis for comparison as long as these factors are kept in mind. Other colony PCR workflows with fungal organisms, primarily using Sanger sequencing, have yielded success rates of 86% and 100% [49,50]. Some colony PCR workflows with bacterial organisms using Sanger sequencing have yielded success rates of 100%, 97.1% and 89.6% [51,53]. There are whole-genome workflows on microbial isolates that yield varying success rates such as 87% and 36% [54,55], where the low success rate is partially attributed to low DNA concentration extracted from the microbes the authors sequenced and additional factors in the quality of the sequence assembly. Others have attempted synthetic long-read sequencing technologies, such as LoopSeq, on fungal and bacterial isolates using conventional isolation protocols, which yielded sequences for the isolates, implying a 100% success rate [56]. Other workflows use non-microbial sample types, for example, one performing both Sanger and Illumina sequencing on DNA extracted from insects using standard protocols, which yielded success rates from 77.4% to 86.1% [57], depending on the gene region that was targeted. Overall, we note that even though there are several factors that prevent a directly principled comparison of the success rates between workflows, in general, the success rate of our workflow is comparable with other sequencing workflows in the literature.

In conclusion, our workflow enables scalable genotyping of microbial isolates with ~90% success. Here, we genotyped microbes and focused on the 16S V4 region, but the workflow can be easily modified to target other genes or regions, such as other 16S variable regions of microbes, the 18S region of plants or the internal transcribed spacer region of fungi. Our primers can also be readily adjusted to include primer pads [11], linkers [11,12] and spacers [13,15]. While there is often a trade-off between the simplicity and performance of a workflow, we have demonstrated a workflow that is relatively simple and conducive to scalability, without major sacrifices to performance. This enables efficient genotyping of large existing collections of isolates for applications such as microbial ecology, clinical microbiology and natural product discovery.

## supplementary material

10.1099/mgen.0.001378 Uncited Supplementary Material 1.

10.1099/mgen.0.001378 Uncited Supplementary Material 2.

## Abbreviations

ASV: amplicon sequence variant
