# Supplementary material for: Scalable genotyping of microbial colonies
Source: Microb Genom. 2025 Mar 19;11(3):001378. doi: 10.1099/mgen.0.001378 (PMC11923105; doi:10.1099/mgen.0.001378)
Supplement: Uncited Supplementary Material 1. [file mgen-11-01378-s001.pdf]

Supplementary Figures

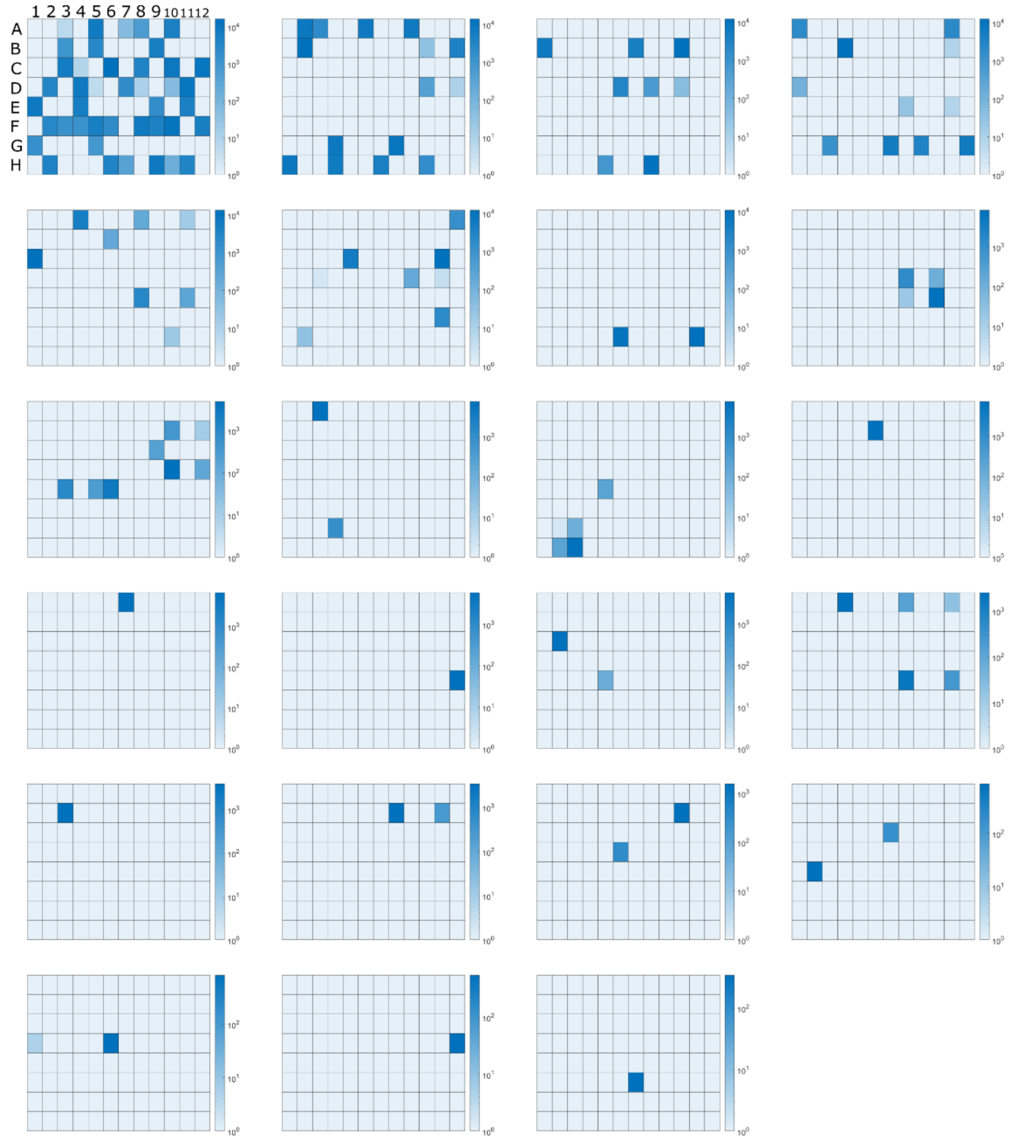

S1 Figure. Read signals for each ASV across the well plate. Each plot corresponds to the read signal of each ASV across the well plate. For example, ASV 12 (row 3, column 4) has a clear signal in well B6, with no signal in any other well. In cases of index hopping, we would expect read signals to hop over to those in the same row or same column, which share one of the indices. In the plots for other ASVs, there are signals appearing in multiple wells. However, we suspect that this is from the selection of colonies cultured from environmental samples, and it is not uncommon to end up with the same taxonomy when randomly picking colonies to sequence. In addition, signal spreading from index hopping would have a more row-like or column-like appearance than what is observed here.

A)

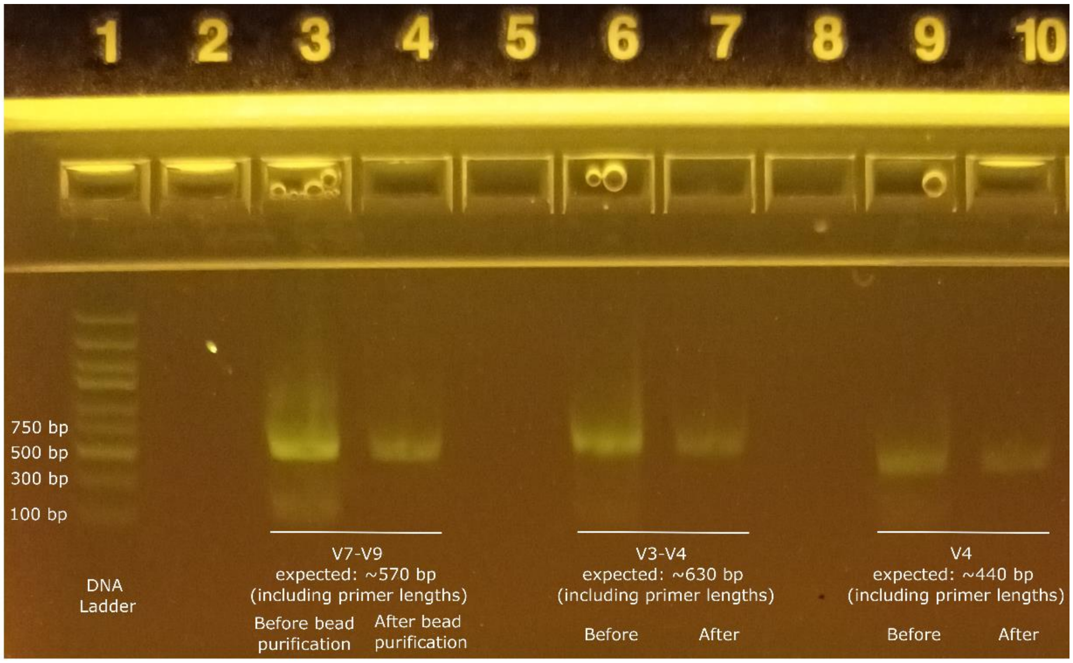

B)

Key

16S V4 | 16S V3 | 16S V7-V9

16S Binding region

Interior barcode

Full Nextera

Exterior barcode

P5/P7

V4

ANCNGANGNGGCGCGTCAGATGTGTATAAGAGACAG CGGCTAATGTGNCAGCCGCCGCGGT
AATACGGAGGGTGCAAGCGTTAATCGGAATAACTGGGCGTAAAGCGCACGCAGGCGGTTTGTT
AAGCCAGATGTGAAAGCCCCGAGCTCAACTCGGGAACTGCATTTGGAACTGGCAAACTAGAGT
CTTGTAGAGGGGGGTAGAATTTCCAGTGTAGCGGTGAAATGCGTAGAGATTGGAAGGAATACCA
GTGGCGAAGGCGGCCCCCTGGACAAAGACTGACGCTCAGGTGCGAAAGCGTGGGGAGCAA
ACAGGATTAGATACCCNGGTAGTCCTGGAAGCACTGTCTCTTATACACATCTCCGAGCCCACGA
GACATCTCAGGATCTCGTATGCCGTCTTCTGCTTGA

V3-V4

NCNGANGNGGCGCGTCGATGTGTATAAGAGACAG CGGCTAATCCTACGGGNGGCTGCAGTG
GGGAATATTGCACAATGGGGGAAACCCTGATGCAGCCATGCCGCGTGTGTGAAGAAGGCCTTC
GGGTTGTAAAGCACTTTCAGTGGTGAGGAAAGGCAGGAACCTAATACGTTTTTGCTGTGACGTTA
ACCACAGAAGAAGCACCGGCTAACTCCGTGCCAGCAGCCGCGGTAATACGGAGGGTGCAAG
CGTTAATCGGAATAACTGGGCGTAAAGCGCACGCAGGCGGTTTGTTAAGCCAGATGTGAAAGC
CCCGAGCTCAACTCGGGAACTGCATTTGGAACTGGCAAACTAGAGTCTTGTAGAGGGGGGTAG
AATTTCCAGTGTAGCGGTGAAATGCGTAGAGATTGGAAGGAATACCAGTGGCGAAGGCGGCCC
CCTGGACAAAGACTGACGCTCAGGTGCGAAAGCGTGGGGAGCAAACAGGATTAGATACCCCG
GTAGTCTGGAAGCACTGTCTCTTATACACATCTCCGAGCCCACGAGACATCTCAGGATCTCGTAT
GCCGTCTTTCTGCTTGA

V7-V9

NCNAANGTCGGCGCGTCGATGTGTATAGAGACAG CGGCTAATCAACGAGCGCAACCCT TGTC
CTTTGTTGCCAGCGGGTAATGCCGGGAACTCAAAGGAGACTGCCGGTGATAAACCGGAGGAA
GGTGGGGACGACGTCAAGTCATCATGGCCCTTACGGGTAGGGCTACACACGTGCTACAATGGC
GCGTACAGAGGGTGGCCAACTCGCGAGAGTGAGCGAATCCCANAAAGCGCGTCGTANTCCG
GATCGGAGTCTGCAACTCGACTCCGTGAAGTCGGAATCGCTAGTAATCGTGGATCAGAATGCCA
CGGTGAATACGTTCCCGGGCCTTGTACACACCGCCCGTCACACCATGGGAGTGGGTTGCTCC
AGAAGTAGATAGCTTAACCTTCGGGAGGGCGTTTACCACGGAGTGATTCATGACTGGGGTGAAG
TCGTAACAAGGTAACCGTATGGAAGCACTGTCTCTTATACACATCTCCGAGCCCACGANACATCT
CAGGATCTCGTATGCCGTCTTTCTGCTTGA

S2 Figure. **Gel electrophoresis and Sanger sequences from targeting 16S V3-V4 and V7-V9 variable regions.** a) Here we perform PCR using primers targeting other combinations of variable regions (16S V3-V4 and V7-V9) on a colony. 16S V4 is also shown alongside for comparison. The gel electrophoresis shows amplification, and the approximate length of the products are as expected. b) The sequence from the products is revealed through Sanger sequencing. The expected adaptor and primer-related regions are present in the product as labeled. The 16S sequences from all three products match to the same taxonomy as revealed through NCBI Blast (*Oceanimonas*). The beginning of the Sanger sequences typically have poor quality and are thus unlabeled/uninterpretable. Overall, we demonstrate that our primers can target other variable regions.

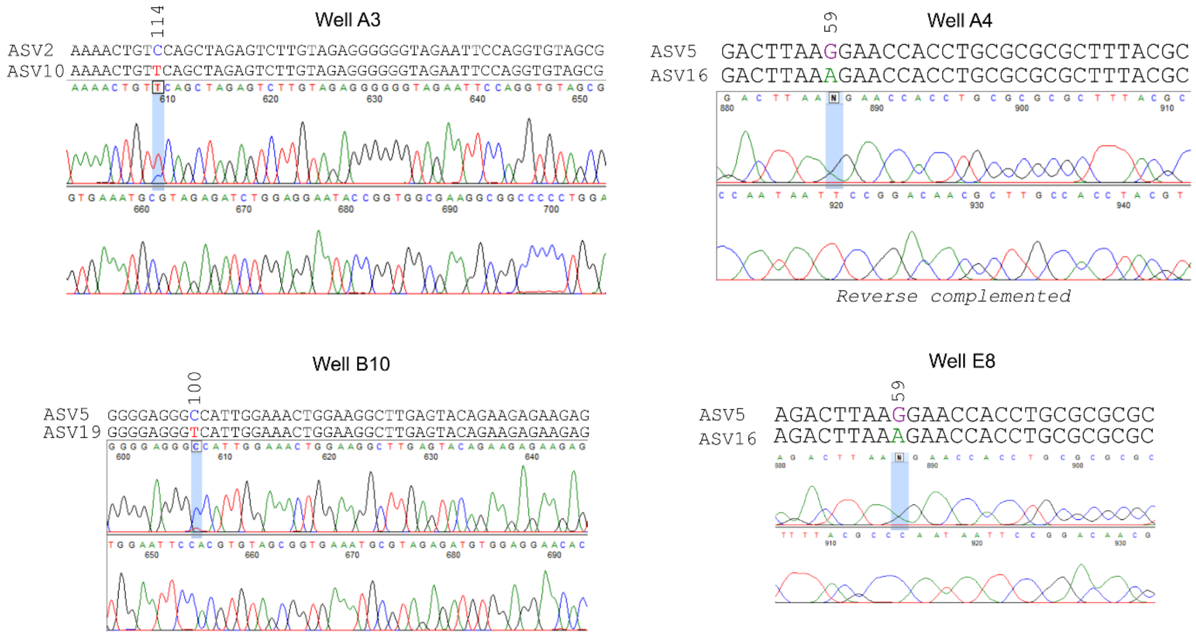

S3 Figure. Wells with ASVs that differ by one base pair appear as a mixed signal in the Sanger sequencing chromatogram. For certain wells, the representative ASVs differ by only 1 base pair. The position of the base pair difference in the ASVs corresponds to the position of the mixed signal in the chromatogram from Sanger sequencing. At this position, the base pairs of the ASVs also match with the base pair signals in the chromatogram. The number above the ASVs indicate the position of this occurrence within the ASVs. The ASVs shown for wells A4 and E8 were reverse complemented to more easily show the comparison with the chromatograms.

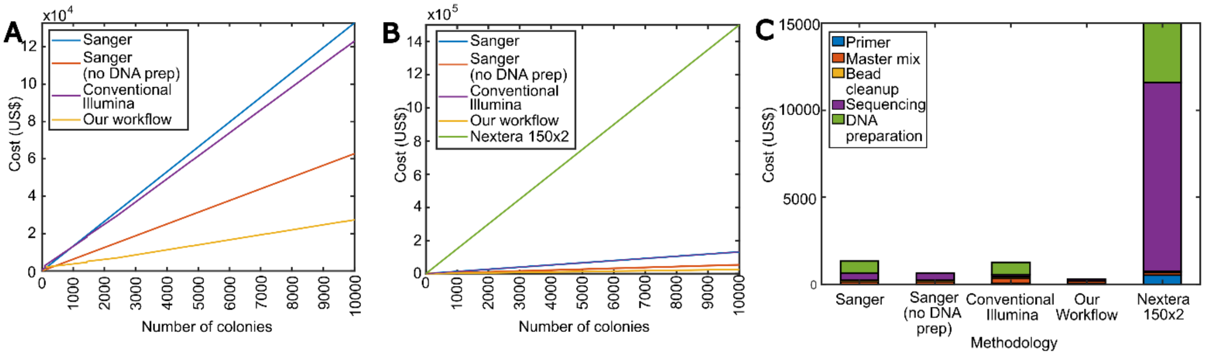

S4 Figure. Alternative cost comparisons between different microbial genotyping workflows. a) Cost comparison using upfront Illumina sequencing costs instead of per colony sequencing cost (Fig. 2B) to compare between the four workflows, The Sanger costs remain unchanged. For the conventional Illumina sequencing and our workflow, the alternative calculation assumes an upfront sequencing cost based on typical Illumina sequencing kits. Assuming 1 million sequencing reads per 96 colonies, even when sequencing below 96 colonies, we include the full cost of the 1 million read MiSeq nano kit (525 US$). Between 96 and 1440 colonies, we include the full cost of the MiSeq v2 kit (1825 US$). Between 1440 and 2400 colonies, we include the full cost of the MiSeq v3 kit (2320 US$). Above 2400 colonies, we assume multiple kits to achieve the minimal upfront sequencing cost. Note that this is a higher cost estimate, and realistically, the unused portion of the sequencing kit can likely be used for other experiments. The break even point between Sanger sequencing with no DNA preparation and our workflow is at 407 colonies. Even with this higher cost estimate, our workflow still scales better in cost than the other workflows. b & c) Adding in a comparison with a workflow using Nextera XT DNA library preparation kit using Illumina 150x2 amplicon sequencing to Fig. 2B and 2C. The Nextera 150x2 workflow has the overall highest cost between the five workflows. Note that even though the cost, especially the sequencing cost, is much higher, the Nextera workflow offers the advantage of being more informative in obtaining sequenced fragments of the genome, as opposed to the sequence of a targeted region. The sequencing cost is under the assumption of sequencing the whole bacterial genome in 300 bp fragments with 100X coverage. In addition, a sizable contribution comes from the cost of the Nextera XT DNA library preparation kit.
